# Health Tract No. 208

**Published:** 1864-06

**Authors:** 


HEALTH TRACT NO. 208.

### RESTLESS NIGHTS.

SOME persons "toss and tumble" half the night and get up in the morning weary, unrefreshed, and dispirited, wholly unfit, either in body or mind, for the duties of the day; they are not only incapacitated for business, but are often rendered so ungracious in their manners, so irritable and fretful, as to spread a gloom and a cloud over the whole household. To be able to go to bed and be in a sound, delicious sleep, an unconscious deliciousness, in five minutes, but enjoyed in its remembrance, is a great happiness, an incalculable blessing, and one for which the most sincere and affectionate thanks should habitually go up to that beneficent Providence which vouchsafes the same through the instrumentalities of a wise and self-denying attention to the laws of our being.

Restless nights as to persons in apparent good health, arise chiefly from, first, an overloaded stomach; second, from worldly care; third, from want of muscular activities proportioned to the needs of the system. Few will have restless nights who take dinner at midday, and nothing after that except a piece of cold bread and butter and a cup or two of some hot drink; any thing beyond that, as cake, pie, chipped beef, doughnuts, preserves, and the like, only tempt nature to eat when there is really no call for it, thus engendering dyspepsia and all its train of evils.

Worldly care. For those who can not sleep from the unsatisfactory condition of their affairs; who feel as if they were going behindhand; or that they are about to encounter great losses, whether from their own remissness, the perfidy of friends, or unavoidable circumstances, we have a deep and sincere sympathy. To such we say, Live hopefully for better days' ahead, and meanwhile strive diligently, persistently, and with a brave heart to that end.

But the more common cause of restless nights is, that exercise has not been taken to make the body tired enough to demand sleep. Few will fail to sleep soundly if the whole of daylight, or as much thereof as will produce moderate fatigue, is spent in steady work in the open air, or on horseback, or on foot. Many spoil all their sleep by attempting to force more on nature than she requires. Few persons will fail to sleep soundly, while they do sleep, if they avoid sleeping in the daytime, will go to bed at a regular hour, and heroically resolve to get up the moment they wake, whether it is at two, four, or six o'clock in the morning. In less than a week, each one will find how much sleep his system requires; thereafter give it that, and no more.

